# Luteolin alleviates methionine–choline-deficient diet-induced non-alcoholic steatohepatitis by modulating host serum metabolome and gut microbiome

**DOI:** 10.3389/fnut.2022.936237

**Published:** 2022-07-28

**Authors:** Wei Guo, Lianxiang Luo, Yan Meng, Wen Chen, Lixiu Yu, Cong Zhang, Zhenpeng Qiu, Peng Cao

**Affiliations:** ^1^Department of Pharmacy, Union Hospital, Tongji Medical College, Huazhong University of Science and Technology, Wuhan, China; ^2^Hubei Province Clinical Research Center for Precision Medicine for Critical Illness, Wuhan, China; ^3^The Marine Biomedical Research Institute, Guangdong Medical University, Zhanjiang, China; ^4^The Marine Biomedical Research Institute of Guangdong Zhanjiang, Zhanjiang, China; ^5^College of Pharmacy, Hubei University of Chinese Medicine, Wuhan, China

**Keywords:** luteolin, non-alcoholic steatohepatitis, metabolomics, gut microbiota, thiamine

## Abstract

**Background and purpose:**

Previous studies have indicated the protective effects of luteolin against non-alcoholic steatohepatitis (NASH), but the definite underlying mechanism still remains unclear. This study aimed to explore the metabolomic and metagenomic signatures of NASH with luteolin supplementation.

**Experimental approach:**

Mice were fed with a methionine–choline-deficient (MCD) diet containing 0.05% luteolin for 6 weeks. NASH severity was determined based on the liver histological observations, serum and hepatic biochemical measurements. Targeted metabolomics was conducted to identify differential metabolites in mice serum. 16S rRNA sequencing was conducted to assess the gut microbiota composition and function in mice colon.

**Results:**

In detail, luteolin treatment significantly alleviated MCD diet-induced hepatic lipid deposition, liver function damage, and oxidative stress. Targeted plasma metabolomics revealed that 5-hydroxyindole, LPE (0:0/22:5), indole 3-phosphate, and N-phenylacetylphenylalanine were remarkably elevated, and homogentisic acid, thiamine, KN-93, PC (16:1e/8, 9-EpETE), carnitine C9:1-OH, FFA (18:4) and carnitine C8:1 were significantly decreased in NASH group as compared to normal group, which could be profoundly reversed after luteolin treatment. 16S rRNA sequencing indicated that luteolin supplementation significantly increased *Erysipelatoclostridium* and *Pseudomonas* as well as decreased *Faecalibaculum* at genus level. Most importantly, a negative association between thiamine and *Faecalibaculum* was observed based on Spearman's correlation analysis, which may play an important role in the preventive effects of luteolin against NASH.

**Conclusion:**

Collectively, luteolin may alleviate the NASH by modulating serum metabolome and gut microbiome, which supports its use as a dietary supplement for NASH prevention.

## Introduction

As the primary form of chronic liver diseases, non-alcoholic fatty liver disease (NAFLD) is comprised of a broad spectrum of liver pathologies from steatosis and steatohepatitis to fibrosis, cirrhosis, and hepatocellular carcinoma ultimately without a history of excessive alcohol consumption (1). Non-alcoholic steatohepatitis (NASH) is a severe form of NAFLD characterized by hepatocellular ballooning, lobular inflammation, and fibrosis. It has been reported that up to 30% of the adult population are affected by NAFLD and almost 25% of NAFLD individuals develop NASH, which is projected to be the commonest indication for liver transplantation over the next decade (2). NASH is a multifactorial disease with complex pathogenesis of “multiple hit” hypothesis, including lipotoxicity, insulin resistance, endoplasmic reticulum stress, mitochondrial dysfunction, inflammation, altered regulation of innate immunity, gut–liver axis, and other factors. Although some lipid-lowering agents, insulin sensitizers, and antioxidants have been developed, currently, no first-line drug has been approved for NASH prevention or therapy by Food and Drug Administration (3). Therefore, it is urgent to develop safe and effective options to prevent the progression of NASH.

The human intestine harbors 500–1,500 different kinds of microorganisms, which encode more than three million genotypes at a ratio of 100 times the host genotypes and possess critical roles in host physiology (4). Recent indisputable evidence suggests that gut microbiota play the essential functions in the pathogenesis of NAFLD and its progression to NASH (5). Various mechanisms of association between gut dysbiosis and NASH pathology have been put forward (6). The disruption of the gut barrier function allows for the translocation of microbial components, such as lipopolysaccharide, into the liver *via* the portal circulation. These microbial components in liver stimulate several stress signaling pathways and thus induce hepatic lipid synthesis and inflammatory response (7). Besides, various gut microbiota-derived metabolisms, such as short-chain fatty acids (SCFAs), bile acids, and ethanol, are the important modulators to modulate NASH susceptibility (8). In general, the modulation of the gut microbiota and its metabolites by diet standardization, or probiotics, is a potential therapeutic for NASH.

As an essential complement to the newest “omics” sciences, metabolomics focuses on the systematic study of metabolome characterized by the small exogenous and endogenous molecule metabolites of cells, biofluids, or tissues in disease states or in response to drug interventions (9). The metabolome also contains various metabolites associated with nutrient ingestion, digestion, and absorption by the gastrointestinal tract and gut microbiota, such as undigested food residues (mucopolysaccharides, fiber, etc.) and small molecules (amino acids, organic acids, sugars, etc.), which contributes to the investigation on the role of gut microbiota. Metabolomics is widely applied for disease monitoring and drug discovery, as well as pharmacodynamic and therapeutic evaluation (10). A number of metabonomic studies provided evidence that disorders in the metabolisms of glucose, lipids, bile acids, and amino acids were associated with NAFLD or NASH (8).

As a long-standing concept in traditional Chinese medicine, “Medicine-food homology” has evoked a tremendous resurrection recently. Luteolin (3',4',5,7-tetrahydroxyflavone) is a natural flavonoid widely derived from fruits, vegetables, and herbs (11). Luteolin processes a variety of biological activities, such as anti-inflammation, anti-oxidative stress, anti-bacteria, anti-virus, and anti-tumor (12, 13). Recently, its protective effects against NAFLD or NASH have also been suggested. Kwon et al. found that luteolin inhibited insulin resistance and prevented diet-induced liver adipocyte fibrosis through toll-like receptor signaling (14). Sun et al. observed that luteolin supplementation enriched gut bacterial species and reduced intestinal permeability and plasma lipopolysaccharide, which prevented the progression of simple steatosis to NASH (15). These studies underscored the regulation of gut microbiome in the protective effects of luteolin against NAFLD or NASH. However, the detailed underlying mechanisms of luteolin in preventing NASH, especially microbiota-derived metabolomic signatures of luteolin, still need to be further investigated.

In our study, mice were fed with a methionine–choline-deficient (MCD) diet to induce NASH. NASH severity was determined based on liver histological observations, serum and hepatic biochemical measurements. Targeted metabolomics and 16S rRNA sequencing were conducted to profile the serum metabolome and gut microbiome, respectively. This study provided experimental evidence that luteolin alleviated MCD diet-induced NASH by modulating serum metabolome and gut microbiome, suggesting luteolin to be a promising dietary supplement for NASH prevention.

## Methods

### Materials and reagents

The powder of luteolin was obtained from Aladdin Bio-Chem Technology Co., Ltd. (Shanghai, China). MCD diet, customized MCD diet containing luteolin (0.05%, w/w), and isocaloric AIN93G diet were purchased from Medicence Lab Animal Diets Co., Ltd. (Jiangsu, China). Commercial kits of alanine aminotransferase (ALT), aspartate transaminase (AST), total cholesterol (TC), and triglyceride (TG) were bought from Nanjing Jiancheng Bioengineering Institute (Nanjing, China). All other experimental materials were commercially available.

### Animal experiments

The 6-week-old SPF-grade male C57BL/6J mice were bought from the Center for Experimental Animal Research (Hubei, China) and maintained in an SPF-level room (25°C, 12-h light/dark cycle). After acclimation for 1 week, they were randomly divided into three groups, including: (1) Normal group: mice fed with an isocaloric AIN93G diet for 6 weeks; (2) NASH group: mice fed with an MCD diet for 6 weeks; (3) Luteolin group: mice fed with a customized MCD diet containing luteolin for 6 weeks. At the end of the 6th week, the blood of each mouse was collected from the orbital sinus and then separated by centrifugation (Eppendorf Centrifuge 5804R) of 1,000 g for 10 min to obtain the serum. Thereafter, colon contents and liver tissues of each mouse were also collected and transferred to a −80°C refrigerator immediately.

### Targeted metabolomic analysis

The targeted metabolomic analysis was performed as previously described (16). In brief, the serum samples were precipitated with 3 volumes of methanol, vortexed sufficiently, and then centrifuged at 16,000 g for 10 min to remove the precipitate. Next, the supernatant was collected for liquid chromatography-electrospray ionization tandem mass spectrometry (LC-ESI-MS/MS) analysis (UPLC, ExionLC AD; MS, QTRAP^®^ System). A Waters ACQUITY UPLC HSS T3 C18 (1.8 μm, 2.1 × 100 mm) column was employed to separate the endogenous metabolites by a gradient elution within 15 min. Signals for metabolites were acquired using a triple quadrupole-linear ion trap mass spectrometer (QTRAP) in positive and negative ion modes. Data were processed using Analyst 1.6.3 software (Sciex).

### 16S rRNA diversity sequencing

First, genomic DNA extraction and PCR amplification were conducted as follows: the genomic DNA of each sample was extracted using the cetyltrimethylammonium bromide (CTAB) method (17), and the purity of DNA was detected by agarose gel electrophoresis. Diluted genomic DNA (1 ng/μl) was then used as the template for PCR amplification using Phusion^®^ High-Fidelity PCR Master Mix with GC Buffer (New England Biolabs). Second, the qualified PCR products, confirmed by electrophoresis on 2% agarose gel, were purified by magnetic beads. Finally, the 16S rRNA diversity sequencing was performed using the platform of NovaSeq6000 after the construction of a library using TruSeq^®^ DNA PCR-Free Sample Preparation Kit.

### Statistics and multivariate correlation analysis

The data of serum biochemistry indicators were expressed as mean ± SD and then analyzed by two-tailed unpaired Student's *t*-test, in which *p* < 0.05 indicated a statistical significance.

For the results of targeted metabolomic analysis, Orthogonal Projections to Latent Structures-Discriminant Analysis (OPLS-DA) was performed by statistic function prcomp within R software (www.r-project.org). Differential expressed metabolites between groups were determined by VIP >1 and absolute Log2FC (fold change) >1, along with *p* < 0.05. VIP values, score plots, and permutation plots (200 permutations) were extracted from OPLS-DA results and generated using R package MetaboAnalystR. The identified metabolites were annotated using Kyoto Encyclopedia of Genes and Genomes (KEGG) compound database, and the annotated metabolites were subsequently mapped to KEGG pathway database. For a given list of metabolites, significantly enriched pathways were determined by the *p*-value of the hypergeometric test.

For the results of 16S rRNA diversity sequencing, alpha diversity indexes, including observed species, community richness (Chao1 & ACE), and community diversity (Shannon and Simpson), were calculated using QIIME software (version 1.9.1). Normal distribution of the data was assessed using the Shapiro–Wilk test. Then, the R software was employed to calculate the between-group difference using Tukey's test (normal distribution) and Wilcox test (non-normal distribution) of agricolae package.

Spearman's correlation analysis was used to discover the relationship between host serum metabolites and microbiota in colon. The correlation analysis was calculated using the cor function of the R software, and the significance test of the correlation was calculated using the corPvalueStudent function of the WGCNA package of the R software. Spearman's correlation coefficients ranged from −1 to 1, of which a positive value indicated a positive correlation whereas a negative value indicated a negative correlation.

## Results

### Luteolin effectively relieved MCD diet-induced NASH

**Figure 1:**
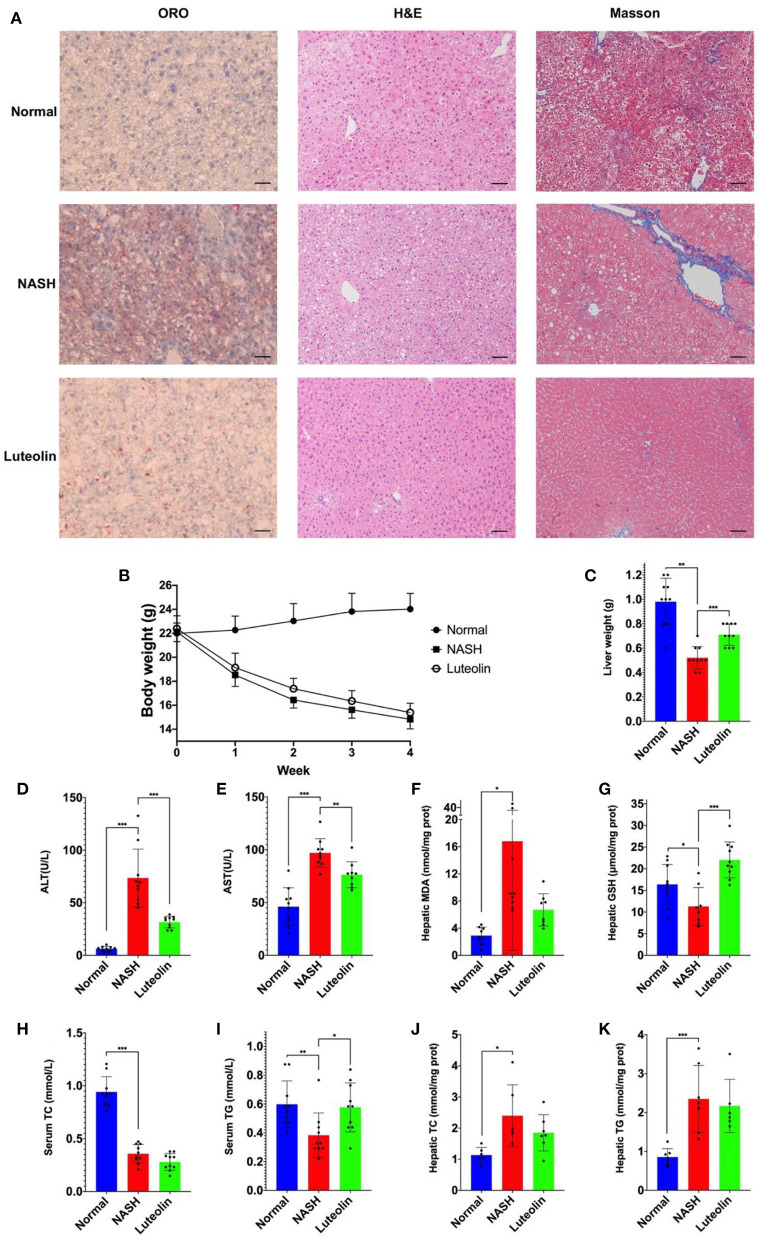
Luteolin effectively relieved NASH (*n* = 6–10 per group). **(A)** Pathological observations (ORO staining, H&E staining, and Masson staining, the scale bar indicated the length of 200 μm); **(B)** body weight monitoring; **(C)** liver weight; **(D)** serum ALT level; **(E)** serum AST level; **(F)** hepatic MDA content; **(G)** hepatic GSH content; **(H)** serum TC content; **(I)** serum TG content; **(J)** hepatic TC content; and **(K)** hepatic TG content (values were expressed as mean ± SD and analyzed by two-tailed unpaired Student's *t*-test, ^***^p < 0.001, ^**^p < 0.01, ^*^p < 0.05).

The protective effect of luteolin toward NASH was identified by liver histological observations, serum and hepatic biochemical measurements. In specific, liver tissue slices were stained by ORO, H&E, and Masson dyes, with the results showing that the livers of NASH mice developed severe lipid accumulation, hepatocyte steatosis, and fibrosis (Figure 1A). Interestingly, luteolin supplementation significantly alleviated all these detrimental histological variations, reversing these lesions to the level nearly identical to that of normal mice.

We also observed the variations in body weight and liver weight in each group. The body weight of mice fed with an MCD diet decreased dramatically compared with the normal mice, but luteolin supplementation slightly prevented weight loss (Figure 1B). Likewise, luteolin significantly reversed the reduction in liver weight induced by MCD diet feeding (Figure 1C).

On the other hand, as displayed in Figures 1D,E, serum ALT and AST levels in NASH mice were dramatically increased compared with the normal control mice (*p* < 0.001). However, luteolin supplementation significantly decreased the abnormal levels of ALT (*p* < 0.001) and AST (*p* < 0.01). Meanwhile, hepatic malondialdehyde (MDA) and glutathione (GSH) contents, which commonly act as the markers of oxidative stress, were also altered significantly in NASH group as compared to Normal group, which could be recovered by luteolin supplementation (Figures 1F,G).

In addition, serum TG and TC contents were significantly reduced when mice were fed with an MCD diet as compared to normal controls (*p* < 0.01). Luteolin could reverse the trend of abnormal TG content (*p* < 0.01), but TC in the serum of NASH mice remained unchanged after luteolin supplementation (Figures 1H,I). Meanwhile, we also measured the hepatic TC and TG contents, and the results showed that luteolin could reduce hepatic lipid accumulation, but they failed to reach a statistical significance (Figures 1J,K).

### Luteolin alleviated the metabolic disorders in MCD diet-fed mice

**Figure 2:**
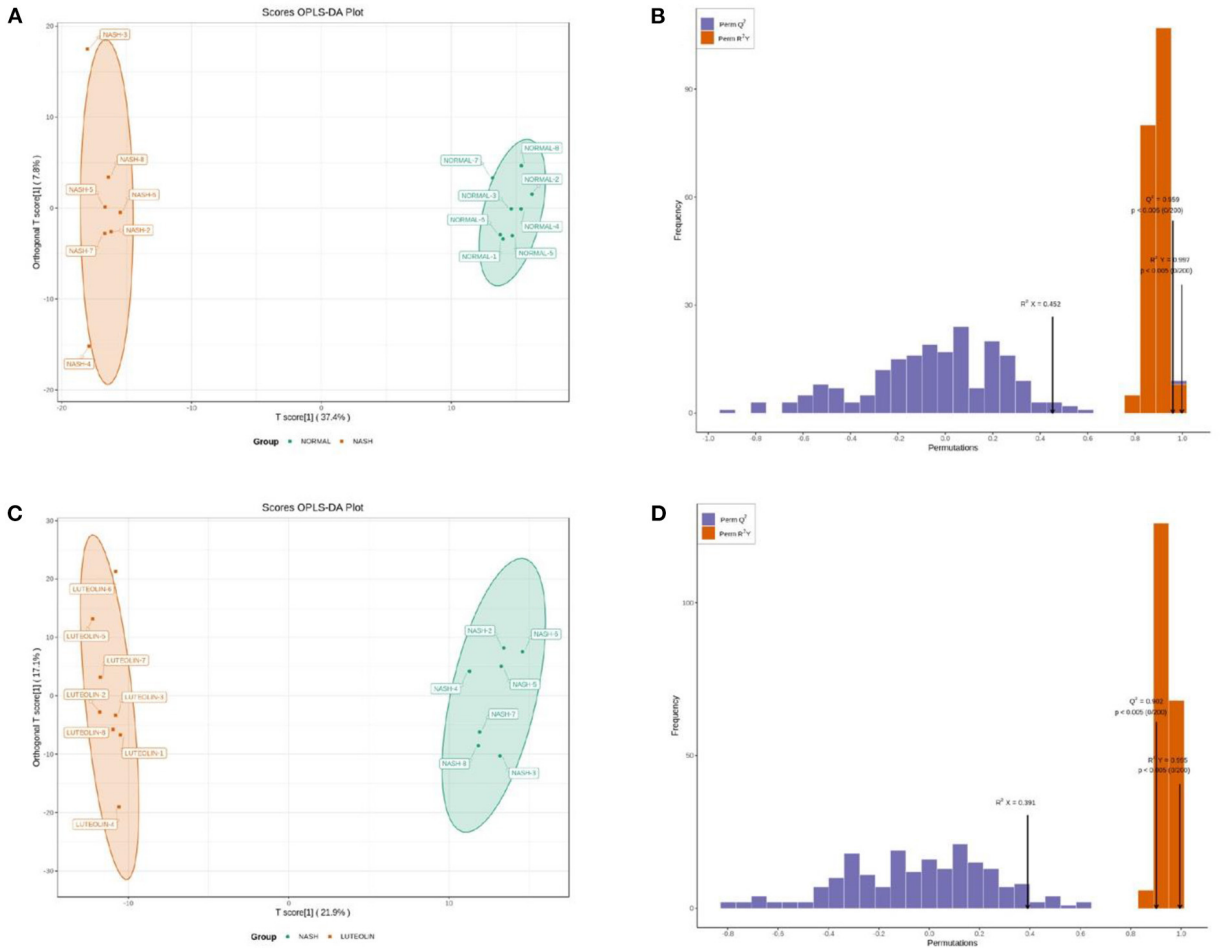
**(A)** OPLAS-DA scoring map between Normal and NASH groups; **(B)** model validation of OPLAS-DA scoring map between Normal and NASH groups; **(C)** OPLAS-DA scoring map between NASH and Luteolin groups; **(D)** model validation of OPLAS-DA scoring map between NASH and Luteolin groups.

Collected serum from mice in different groups was analyzed using a target metabolomic method. First of all, the metabolome data were analyzed according to the OPLS-DA model. As shown in Figures 2A,C, the metabolic profiles were totally separated in different groups (Normal vs. NASH, and NASH vs. Luteolin). In addition, the score map of each model was drawn to further demonstrate the accuracy and efficiency (Figures 2B,D). *R*^2^*Y* and *Q*^2^ values are the important parameters, with a value closer to 1 indicating a more robust model. In this study, the *R*^2^*Y* and *Q*^2^ values were 0.959 (*p* < 0.005) and 0.997 (*p* < 0.005), respectively, in the model Normal vs. NASH. On the other hand, the *R*^2^*Y* and *Q*^2^ values of model NASH vs. Luteolin were 0.995 (*p* < 0.005) and 0.902 (*p* < 0.005), respectively. These results demonstrated the satisfactory abilities of the OPLS-DA models to discriminate against different groups.

**Figure 3:**
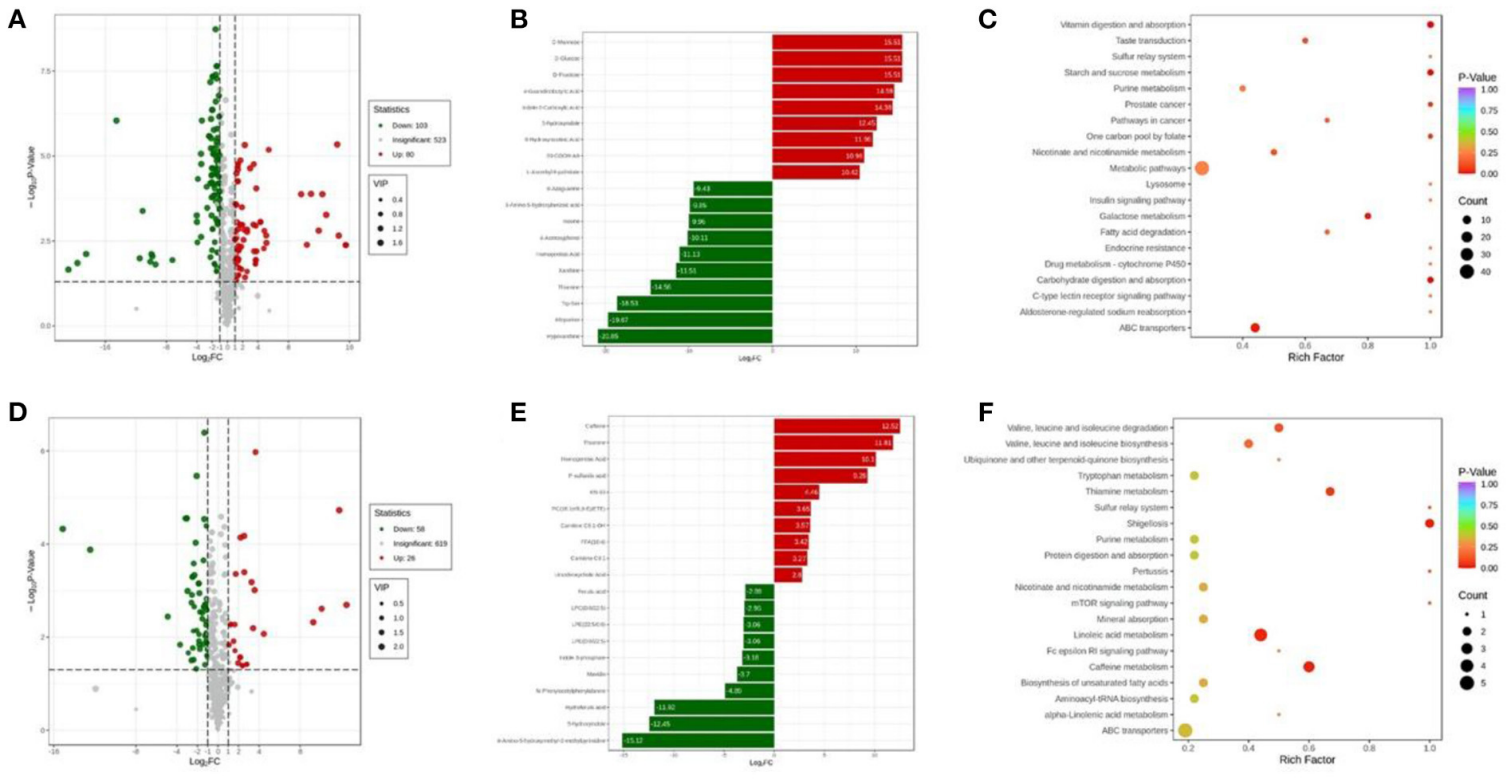
**(A)** Volcano map of differentially expressed metabolites between Normal group and NASH group; **(B)** the top 20 differentially expressed metabolites with the largest fold changes between Normal group and NASH group; **(C)** the enrichment analysis of differentially expressed metabolites between Normal group and NASH group; **(D)** volcano map of differentially expressed metabolites between NASH group and Luteolin group; **(E)** the top 20 differentially expressed metabolites with the largest fold changes between NASH group and Luteolin group; **(F)** the enrichment analysis of differentially expressed metabolites between NASH group and Luteolin group. (*n* = 6–8 per group).

Further, volcano maps were plotted to display the differential expressed metabolites among distinct groups based on a combined screening according to VIP values, fold changes, and *p*-values. As shown in Figure 3A, a total of 183 kinds of metabolites were found differentially between Normal group and NASH group. More clearly, Figure 3B shows the most significant differential expressed metabolites, in which the contents of D-mannose, D-glucose, D-fructose, 4-guanidinobutyric acid, Indole-2-carboxylic acid, 5-hydroxyindole, 6-hydroxynicotinic acid, 20-COOH-AA, and L-ascorbyl 6-palmitate were increased the most in the serum of NASH group compared with that of Normal group, whereas the top 10 decreased metabolites in NASH group compared with its counterpart were hypoxanthine, allopurinol, Trp-Ser, thiamine, xanthine, homogentisic acid, 4-acetoxyphenol, inosine, 3-amino-5-hydroxybenzoic acid, and 8-azaguanine. The differential expressed metabolites between NASH group and Normal group could be enriched in the pathways of vitamin digestion and absorption, starch and sucrose metabolism, carbohydrate digestion and absorption, etc. (Figure 3C).

On the other hand, the levels of 84 kinds of detected metabolites were significantly different between NASH group and Luteolin group (Figure 3D). Among them, caffeine, thiamine, homogentisic acid, P-sulfanilic acid, KN-93, PC (16:1e/8,9-EpETE), carnitine C9:1-OH, FFA (18:4), carnitine C8:1, and ursodeoxycholic acid were elevated the most in Luteolin group compared with NASH group, whereas, 4-amino-5-hydroxymethyl-2-methylpyrimidine, 5-hydroxyindole, hydroferulic acid, N-phenylacetylphenylalanine, mavidin, indole 3-phosphate, LPE (0:0/22:5)/ LPE (22:5/0:0), LPC (0:0/22:5), and ferulic acid were decreased the most in Luteolin group compared with the counterpart group (Figure 3E). The differentially expressed metabolites between Luteolin group and NASH group could be enriched in the pathways of shigellosis, thiamine metabolism, caffeine metabolism, valine, leucine, and isoleucine degradation/biosynthesis, linoleic acid metabolism, etc. (Figure 3F).

### The differential expressed metabolites among normal, NASH, and luteolin groups

**Figure 4:**
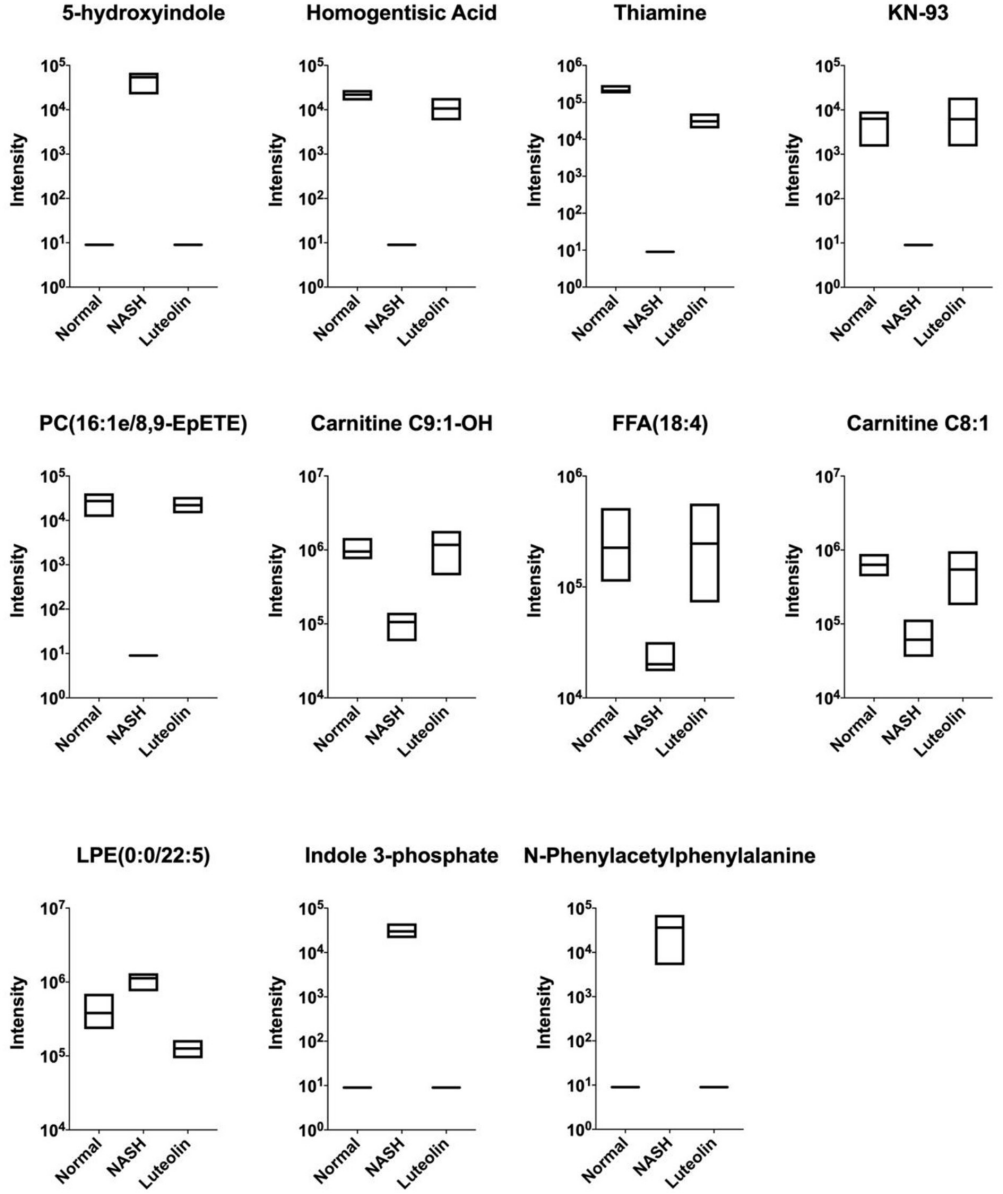
Shared differentially expressed metabolites among Normal, NASH, and Luteolin groups, displayed by boxplots (the upper and lower edges of the box plot were the maximum and minimum values, respectively, while the middle line represented the median value) (*n* = 6–8 per group).

As mentioned above, there existed lots of differentially expressed metabolites between NASH group and Normal/Luteolin groups. Furthermore, we found significant differences in some metabolites between the three groups (Figure 4). In specific, on the one hand, the contents of 5-hydroxyindole, LPE (0:0/22:5), indole 3-phosphate, and N-phenylacetylphenylalanine were remarkably elevated in NASH group when compared to Normal group, which could be significantly reduced after luteolin supplementation. On the other hand, the levels of homogentisic acid, thiamine, KN-93, PC (16:1e/8,9-EpETE), carnitine C9:1-OH, FFA (18:4), and carnitine C8:1 were significantly lowed when mice were fed with an MCD diet in NASH group as compared to the mice fed with a regular diet in Normal group. However, luteolin supplementation could reverse the abnormal levels of them, which might account for the protective effects of luteolin.

### Luteolin modulated gut microbiota composition in MCD diet-fed mice

**Figure 5:**
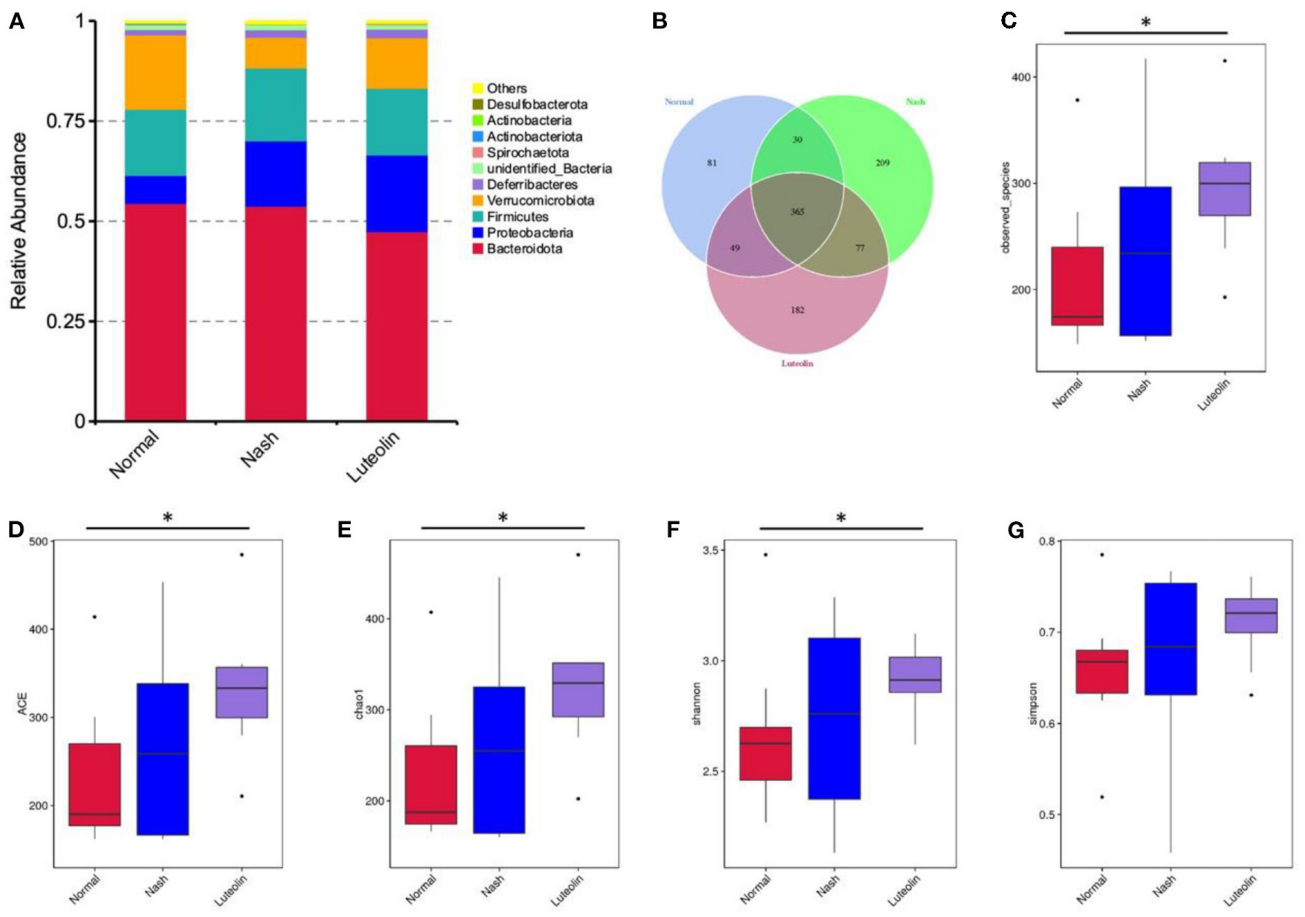
Changes in gut microbiome composition among different groups. **(A)** Histogram of relative abundance of species at the phylum level; **(B)** Venn diagram; **(C)** boxplot of observed species; **(D)** boxplot of ACE index; **(E)** boxplot of Chao1 index; **(F)** boxplot of Shannon index; **(G)** boxplot of Simpson index. (^*^p < 0.05, *n* = 6–8 per group).

The intestinal contents of each mouse were analyzed using the 16S rDNA amplicon sequencing. The microbial composition at phylum level is displayed in Figure 5A, and it clearly showed that *Verrucomicrobiota* were reduced in NASH group compared to Normal group, which was reversed in Luteolin group. A Venn diagram also displayed the detected operational taxonomic units (OTUs) in different groups (Figure 5B). Moreover, the observed species and alpha diversity analysis, including Shannon, Simpson, Chao1, and ACE indexes, are shown in Figures 5C–G. The results demonstrated that the number of microbial species was increased, as well as the alpha diversity indexes were improved in NASH group compared with Normal group. Interestingly, luteolin supplementation further increased the microbial richness in mice fed with an MCD diet.

### Differential gut microbiome species between NASH and luteolin groups at different taxonomic levels

**Figure 6:**
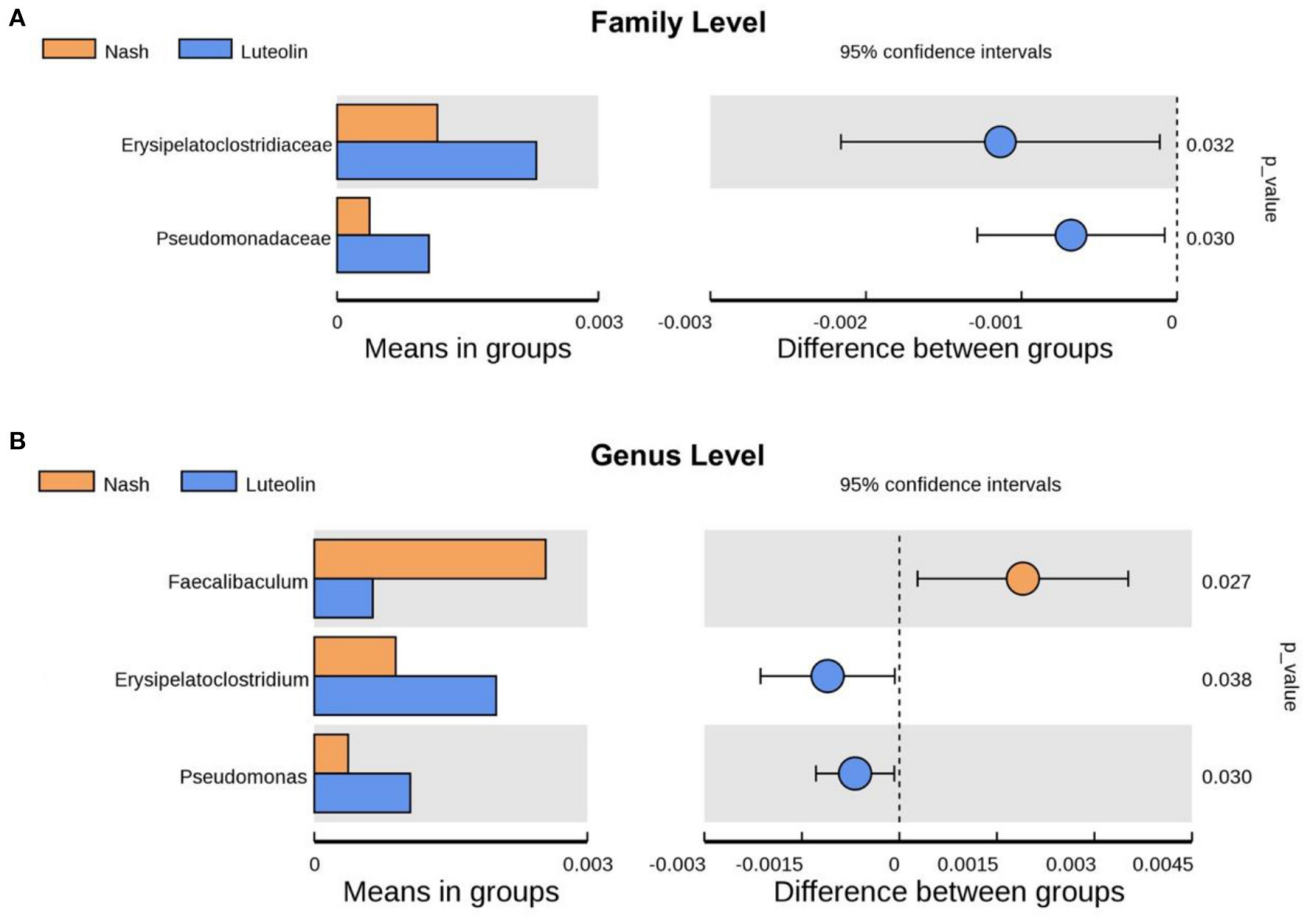
Differential gut microbiome species between NASH and Luteolin groups at **(A)** family level and **(B)** genus level (*n* = 6–8 per group).

To find the different species between Luteolin group and NASH group at various classification levels (phylum, class, order, family, genus, and species), a *t*-test was performed to find out the species with significant differences (*p* < 0.05). At family level, the levels of *Erysipelatoclostridiaceae* and *Pseudomonadaceae* were increased in Luteolin group compared with NASH group (Figure 6A). After further refining the level to genus, the contents of *Erysipelatoclostridium* and *Pseudomonas* were still increased in Luteolin group, which was consistent with the results at family level (Figure 6B). Meanwhile, the genus level of *Faecalibaculum* was significantly decreased after luteolin supplementation.

### Correlation of gut microbiota and metabolic phenotype in MCD diet-fed mice after luteolin supplementation

**Figure 7:**
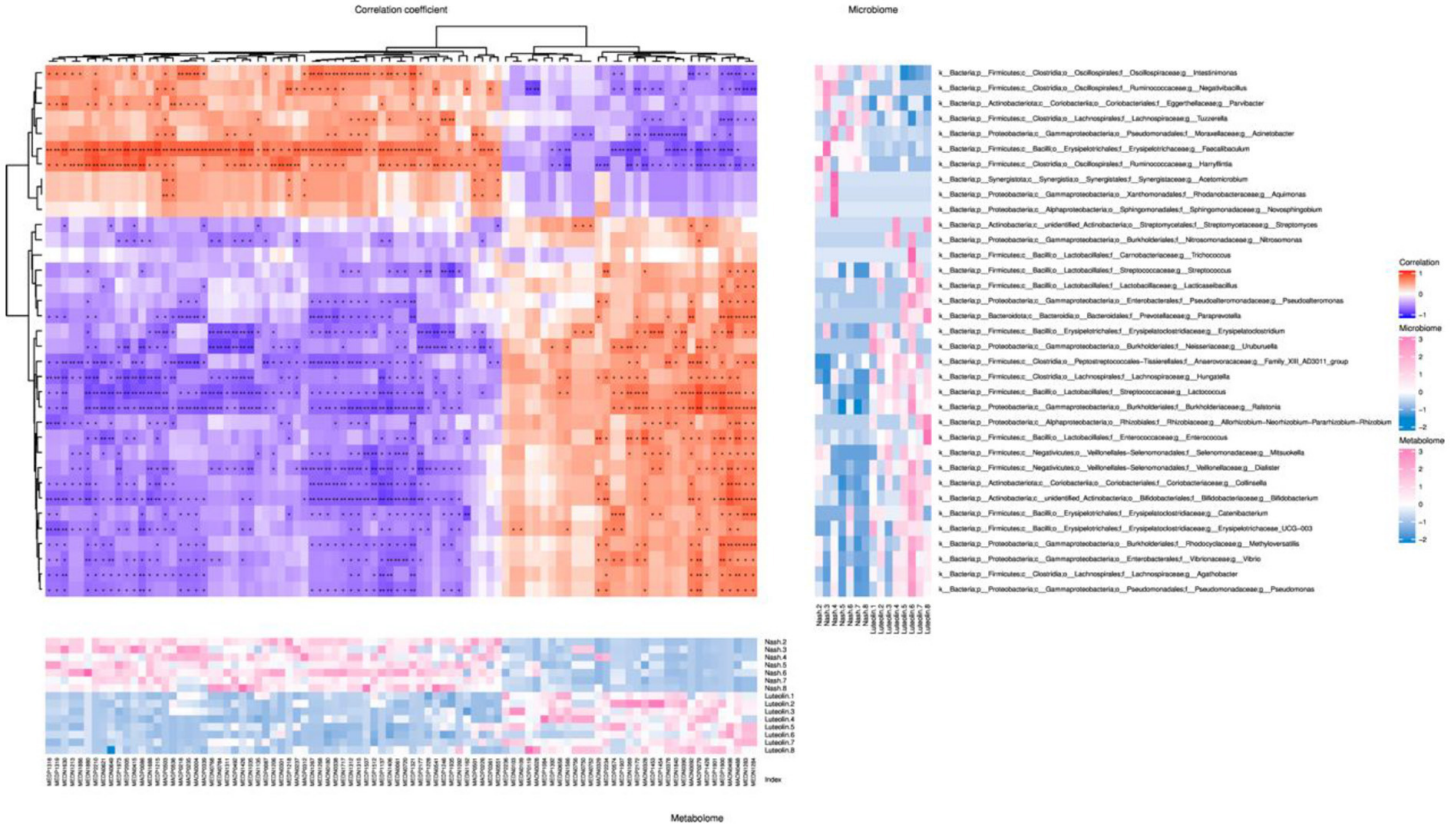
Spearman's correlation cluster heatmap of differential gut microbiome and differential metabolites at genus level (The central heatmap showed the magnitude of the Spearman's correlation between differential microbes and differential metabolites. ^*^p < 0.05, ^**^p < 0.01, *n* = 6–8 per groups. The abscissa represented metabolites, and the ordinate represented microbes. The heatmap on the right showed the abundance of microbes at genus levels, and the heatmap on the bottom showed the abundance of metabolites.).

Spearman's correlation analysis was performed on differential gut microorganisms at genus level and differential host serum metabolites (Figure 7). Spearman's coefficient *r* > 0.8 or *r* < −0.8, together with a *p*-value below 0.05, was set as the criteria of significant correlation. The results showed many relevant microbiota/differential metabolite combinations, with thiamine and *Faecalibaculum* appearing the most times as differential metabolite and differential genus-level microbiota, respectively, among all related combinations.

**Figure 8:**
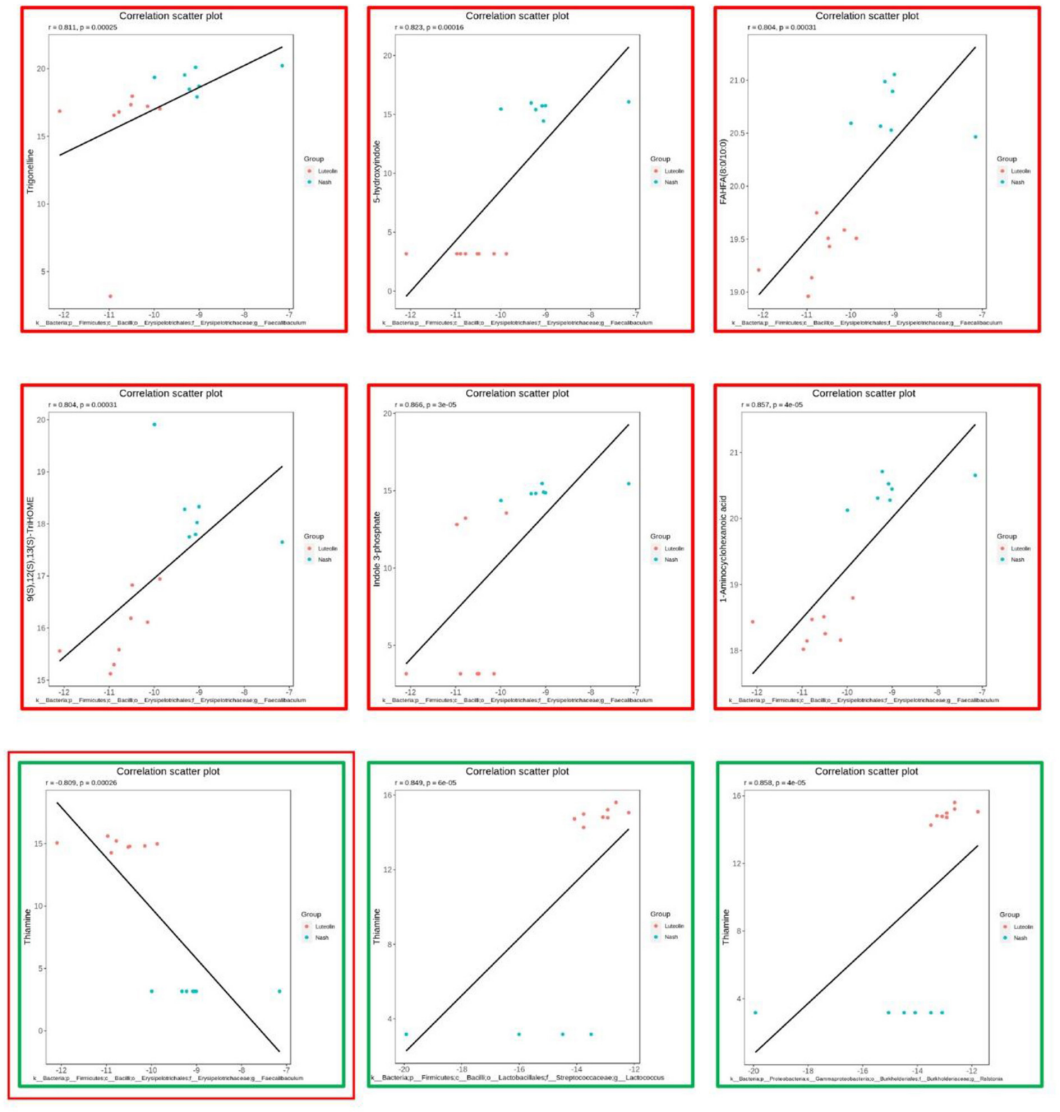
The most significantly correlated microbes and metabolites (*n* = 6–8 per group).

The scatter plots were drawn to show the combinations, including thiamine and *Faecalibaculum* (Figure 8). Specifically speaking, thiamine was significantly positively correlated with *Lactococcus* and *Ralstonia*, yet negatively associated with *Faecalibaculum*. In addition, *Faecalibaculum* was also positively correlated with trigonelline, 9(S),12(S),13(S)-TriHOME, FAHFA (8:0/10:0), indole 3-phosphate, 1-aminocyclohexanoic acid, and 5-hydroxyindole.

## Discussion

As a severe form of NAFLD, NASH is a multifactorial disease with complex pathogenesis. Currently, no first-line drug has been approved for NASH prevention or therapy. Luteolin is a natural flavonoid with biological activities, and its protective effects against NASH have been suggested recently. In this study, LC-ESI-MS/MS-based metabonomics coupled with 16S rRNA gene sequencing was conducted. To our best knowledge, this is the first study on the accomplishment of multi-omics-based mechanistic elucidation of luteolin dietary supplementation for NASH prevention.

Several obesogenic and nutrient-deficient diet-induced NASH animal models, such as high-fat diet (HFD) and MCD diet, have been built to recapitulate the etiology, natural history, histopathology, and progression of NASH. Despite the known disparities in the metabolic profile of human NASH, MCD dietary mice NASH model better simulates the pathological manifestations of human NASH when compared to other dietary animal NASH models. This model has been widely applied for preclinical drug development for NASH (18). In this study, we successfully built a NASH model by feeding mice with an MCD diet for 6 weeks, as evidenced by hepatic lipid deposition (increased hepatic TC, TG, and liver histological observations), liver function damage (elevated serum ALT and AST levels), and oxidative stress (increased hepatic MDA and decreased GSH levels) in MCD diet-fed mice. These histologic observations and biochemical measurements were remarkably reversed by luteolin supplementation, indicating that luteolin could attenuate hepatic steatosis and ameliorate liver function and oxidative stress in MCD diet-induced NASH mice model.

To investigate the underlying mechanism of how luteolin worked against NASH, an LC-ESI-MS/MS-based serum metabonomics was conducted. Based on the OPLS-DA models, different metabolic profiles among the three experimental groups were observed. In specific, 5-hydroxyindole, LPE (0:0/22:5), indole 3-phosphate, and N-phenylacetylphenylalanine were remarkably elevated, and homogentisic acid, thiamine, KN-93, PC (16:1e/8, 9-EpETE), carnitine C9:1-OH, FFA (18:4), and carnitine C8:1 were significantly decreased in NASH group as compared to normal group, which could be profoundly reversed after luteolin supplementation. 5-hydroxyindole and indole 3-phosphate belong to indole derivatives, which were found to increase in MCD diet-fed model. A previous study reported that metabolic dysregulations in indole compounds were extremely related to NAFLD progression (19). MCD diet increased LPE (0:0/22:5) and decreased PC (16:1e/8, 9-EpETE) and FFA (18:4) levels, which belong to lipid metabolism. The alteration in lipid metabolism, especially phosphatidylcholine metabolites, was suggested to be associated with liver injury, lipid peroxidation, and inflammation and thus plays an essential role in the pathogenesis of NASH (8, 20). Carnitine C9:1-OH and carnitine C8:1 belong to carnitine metabolism, which were observed to decrease in NASH model. Zhao et al. found that carnitine deficiency occurred when mice were given N,N,N-trimethyl-5-aminovaleric acid (TMAVA), which decreased hepatic fatty acid oxidation and increased uptake and liver accumulation of free fatty acids and thus exacerbated HFD-induced fatty liver (21). In addition, two previous studies revealed that patients with NASH could benefit from carnitine supplementation (22, 23). N-phenylacetylphenylalanine was observed to increase in MCD diet-fed model. It has been indicated that amino acid content alterations in metabolic diseases are involved in insulin resistance (24). Homogentisic acid, a tyrosine metabolite, was reported to be associated with chronic liver diseases *via* the regulation of rapid hepatocyte death (25). In our study, luteolin supplementation could reverse the MCD diet-induced abnormal levels of these metabolites mentioned above, which might account for the preventive effects of luteolin against NASH.

Since 90% of microorganisms in the host are located in the colon, the colon contents were chosen to assess the gut microbiota composition and function based on 16S rRNA sequencing (20). In our study, the number of microbial species was increased, and the improvement in alpha diversity indexes was found in MCD diet model. Interestingly, luteolin supplementation further increased the microbial richness in mice fed with an MCD diet. Various studies have revealed that a large number of flavonoids, including luteolin, could elevate the alpha diversity of microbiota in HFD-fed rats (15). Most intestinal microbiota belong to two major phyla, namely, *Firmicutes* and *Bacteroidetes*, followed by two minority phyla, *Actinobacteria* and *Proteobacteria*, and the rest belong to *Verrucomicrobia* and *Fusobacteria* (5). In our study, *Verrucomicrobiota* at phylum level were reduced in NASH group. A similar decrease in *Verrucomicrobiota* was found in mice fed with 12 weeks of HFD (26). Luteolin supplementation attenuated the changes in the gut microbiota structures through increasing the abundance of *Verrucomicrobiota*. Further classification of gut microbiota to the level of genus indicated that luteolin supplementation significantly increased *Erysipelatoclostridium* and *Pseudomonas*, as well as decreased *Faecalibaculum*. *Erysipelatoclostridium*, known to produce SCFAs, was shown to be relevant to hepatic steatosis (8). TMAVA was shown to be associated with liver steatosis, which is metabolized by gut bacteria *Pseudomonas* (21). Altogether, our results indicated that restoration of intestinal bacterial structure contributed to the beneficial effects of luteolin on NASH.

The roles of gut microbiota are remarkably associated with host–microbe metabolic axes (20). To explore the vital intestinal microbiota and the corresponding metabolites that contributed to the protective effects of luteolin against NASH, Spearman's correlation analysis was performed on differential gut microorganisms at genus level and differential host serum metabolites. Our results indicated that thiamine was significantly positively correlated with *Lactococcus* and *Ralstonia*, yet negatively associated with *Faecalibaculum*. Thiamine is a kind of vitamin also called vitamin B1. A recent study revealed that high-dose thiamine supplementation could prevent the development of overnutrition-induced hepatic steatosis (27). In detail, thiamine supplementation increased the glycogen level of the liver and increased the catalytic capacity of hepatic oxidation for carbohydrates and fatty acids. Besides, another recent study also suggested that subjects who consumed a more nutrient-rich and healthy diet, especially in terms of vitamins D and B group, such as thiamine, were at a lower risk of non-alcoholic fatty liver disease when compared to those who followed a nutrient-poor and unhealthy diet (28). Accordingly, it was speculated that the protective effects of luteolin on NASH were partly attributed to higher thiamine excretions. *Lactococcus* was reported to protect mice against Western-style diet-induced metabolic changes (29). *Faecalibaculum*, known as a saccharolytic butyrate producer, is implicated in gut health (30). Patients with NASH or NAFLD were reported to display the differences in *Faecalibaculum* when compared to control cohorts (31). The association between thiamine and these three intestinal species indicated that these species might participate in the preventive effects of luteolin against NASH by regulating thiamine metabolism. Besides, *Faecalibaculum* was also positively correlated with trigonelline, 9(S),12(S),13(S)-TriHOME, FAHFA (8:0/10:0), indole 3-phosphate, 1-aminocyclohexanoic acid, and 5-hydroxyindole. Indole derivatives (indole 3-phosphate and 5-hydroxyindole) are the ligands for aryl hydrocarbon receptor, which is associated with immune system and gut barrier function (32). The association between indole derivatives and *Faecalibaculum* suggested that *Faecalibaculum* might participate in the preventive effects of luteolin against NASH by regulating indole derivatives (indole 3-phosphate and 5-hydroxyindole). Collectively, these correlations suggested that the reduction of *Faecalibaculum* accompanied with the regulation of thiamine and indole metabolisms may play an important role in the protective effects of luteolin against NASH.

Notably, our current study has several limitations. First, although MCD dietary mice NASH model greatly simulates the pathological manifestations of human NASH characterized by inflammatory infiltration and hepatic steatosis, this model is not entirely representative of human NASH due to the absence of characteristics such as insulin resistance and obesity. Hence, further clinical trials of luteolin for NASH are warranted. Second, the regulation of luteolin on gut microbiota needs to be verified *via* fecal microbiota transplantation with germ-free mice. Last but not least, although our results indicated that luteolin modulated the features of the gut microbiota and circulating metabolites in NASH mice, further investigations to confirm the mechanisms that link the gut microbiome and host metabolome alterations are necessary in the future.

## Conclusion

In this study, a combined multi-omics of LC-ESI-MS/MS-based serum metagenomics and 16S rRNA gene sequencing was conducted to investigate the underlying mechanisms of luteolin in the prevention of NASH. Our results indicated that dietary supplementation with luteolin modulated serum metabolome and gut microbiome to alleviate the MCD diet-induced NASH. In conclusion, our work provides a novel insight that luteolin might serve as a promising dietary supplement for NASH prevention.

## Data availability statement

The data presented in the study are deposited in the NCBI repository, accession number: PRJNA850898, ID: 850898, https://www.ncbi.nlm.nih.gov/bioproject/850898.

## Ethics statement

The animal study was reviewed and approved by Institutional Animal Care and Use Committee of Tongji Medical College, Huazhong University of Science and Technology.

## Author contributions

WG: methodology, data curation, and writing. LL and YM: methodology, formal analysis, and data curation. WC and LY: investigation and data. CZ: data curation and statistical analysis. ZQ: supervision and resources. PC: conceptualization, methodology, data curation, editing, and supervision. All authors contributed to the article and approved the submitted version.

## Funding

This study was supported by National Natural Science Foundation of China (Grant Nos. 81903901, 31900919, and U21A20297) and Dawning Program of Wuhan Knowledge Innovation Special Project (Grant No. 2022020801020467).

## Conflict of interest

The authors declare that the research was conducted in the absence of any commercial or financial relationships that could be construed as a potential conflict of interest.

## Publisher's note

All claims expressed in this article are solely those of the authors and do not necessarily represent those of their affiliated organizations, or those of the publisher, the editors and the reviewers. Any product that may be evaluated in this article, or claim that may be made by its manufacturer, is not guaranteed or endorsed by the publisher.

## References

[1] Huang DQ El-Serag HB Loomba R. Global epidemiology of NAFLD-related HCC: trends, predictions, risk factors and prevention. Nat Rev Gastroenterol Hepatol. (2021) 18:223–38. 10.1038/s41575-020-00381-6 33349658 PMC8016738

[2] Musso G Cassader M Gambino R. Non-alcoholic steatohepatitis: emerging molecular targets and therapeutic strategies. Nat Rev Drug Discov. (2016) 15:249–74. 10.1038/nrd.2015.3 26794269

[3] Foerster F Gairing SJ Muller L Galle PR. NAFLD-driven HCC: Safety and efficacy of current and emerging treatment options. J Hepatol. (2022) 76:446–57. 10.1016/j.jhep.2021.09.007 34555422

[4] Khan MT Nieuwdorp M Backhed F. Microbial modulation of insulin sensitivity. Cell Metab. (2014) 20:753–60. 10.1016/j.cmet.2014.07.006 25176147

[5] Park JW Kim SE Lee NY Kim JH Jung JH Jang MK. Role of Microbiota-Derived Metabolites in Alcoholic and Non-Alcoholic Fatty Liver Diseases. Int J Mol Sci. (2021) 23. 10.3390/ijms23010426 35008852 PMC8745242

[6] Knudsen C Neyrinck AM Lanthier N Delzenne NM. Microbiota and nonalcoholic fatty liver disease: promising prospects for clinical interventions? Curr Opin Clin Nutr Metab Care. (2019) 22:393–400. 10.1097/MCO.0000000000000584 31219825

[7] Untersmayr E Brandt A Koidl L Bergheim I. The Intestinal Barrier Dysfunction as Driving Factor of Inflammaging. Nutrients. (2022) 14. 10.3390/nu14050949 35267924 PMC8912763

[8] Gong MJ Zhu CY Zou ZJ Han B Huang P. Therapeutic potential of puerarin against methionine-choline-deficient diet-induced non-alcoholic steatohepatitis determined by combination of (1)H NMR spectroscopy-based metabonomics and 16S rRNA gene sequencing. J Pharm Biomed Anal. (2021) 197:113964. 10.1016/j.jpba.2021.113964 33601157

[9] Zampieri M Sekar K Zamboni N Sauer U. Frontiers of high-throughput metabolomics. Curr Opin Chem Biol. (2017) 36:15–23. 10.1016/j.cbpa.2016.12.006 28064089

[10] Guo W Jiang C Yang L Li T Liu X Jin M. Quantitative metabolomic profiling of plasma, urine, and liver extracts by (1)H NMR spectroscopy characterizes different stages of atherosclerosis in hamsters. J Proteome Res. (2016) 15:3500–10. 10.1021/acs.jproteome.6b00179 27570155

[11] Liu X Sun R Li Z Xiao R Lv P Sun X. Luteolin alleviates non-alcoholic fatty liver disease in rats via restoration of intestinal mucosal barrier damage and microbiota imbalance involving in gut-liver axis. Arch Biochem Biophys. (2021) 711:109019. 10.1016/j.abb.2021.109019 34478730

[12] Ganai SA Sheikh FA Baba ZA Mir MA Mantoo MA Yatoo MA. Anticancer activity of the plant flavonoid luteolin against preclinical models of various cancers and insights on different signalling mechanisms modulated. Phytother Res. (2021) 35:3509–32. 10.1002/ptr.7044 33580629

[13] Caporali S De Stefano A Calabrese C Giovannelli A Pieri M Savini I. Anti-inflammatory and active biological properties of the plant-derived bioactive compounds luteolin and luteolin 7-glucoside. Nutrients. (2022) 14. 10.3390/nu14061155 35334812 PMC8949538

[14] Kwon EY Choi MS. Luteolin targets the toll-like receptor signaling pathway in prevention of hepatic and adipocyte fibrosis and insulin resistance in diet-induced obese mice. Nutrients. (2018) 10. 10.3390/nu10101415 30282902 PMC6213163

[15] Sun WL Yang JW Dou HY Li GQ Li XY Shen L. Anti-inflammatory effect of luteolin is related to the changes in the gut microbiota and contributes to preventing the progression from simple steatosis to nonalcoholic steatohepatitis. Bioorg Chem. (2021) 112:104966. 10.1016/j.bioorg.2021.104966 33991837

[16] Cao P Wu S Guo W Zhang Q Gong W Li Q. Precise pathological classification of non-small cell lung adenocarcinoma and squamous carcinoma based on an integrated platform of targeted metabolome and lipidome. Metabolomics. (2021) 17:98. 10.1007/s11306-021-01849-5 34729658

[17] Yu G Hatta A Periyannan S Lagudah E Wulff BBH. Isolation of wheat genomic DNA for gene mapping and cloning. Methods Mol Biol. (2017) 1659:207–13. 10.1007/978-1-4939-7249-4_18 28856653

[18] Hansen HH Feigh M Veidal SS Rigbolt KT Vrang N Fosgerau K. Mouse models of nonalcoholic steatohepatitis in preclinical drug development. Drug Discov Today. (2017) 22:1707–18. 10.1016/j.drudis.2017.06.007 28687459

[19] Yu JS Youn GS Choi J Kim CH Kim BY Yang SJ. Lactobacillus lactis and Pediococcus pentosaceus-driven reprogramming of gut microbiome and metabolome ameliorates the progression of non-alcoholic fatty liver disease. Clin Transl Med. (2021) 11:e634. 10.1002/ctm2.634 34965016 PMC8715831

[20] Zhang Z Chen X Cui B. Modulation of the fecal microbiome and metabolome by resistant dextrin ameliorates hepatic steatosis and mitochondrial abnormalities in mice. Food Funct. (2021) 12:4504–18. 10.1039/D1FO00249J 33885128

[21] Zhao M Zhao L Xiong X He Y Huang W Liu Z. TMAVA, a Metabolite of intestinal microbes, is increased in plasma from patients with liver steatosis, inhibits gamma-butyrobetaine hydroxylase, and exacerbates fatty liver in mice. Gastroenterology. (2020) 158:2266–81. e2227. 10.1053/j.gastro.2020.02.033 32105727

[22] Malaguarnera M Gargante MP Russo C Antic T Vacante M Malaguarnera M. L-carnitine supplementation to diet: a new tool in treatment of nonalcoholic steatohepatitis–a randomized and controlled clinical trial. Am J Gastroenterol. (2010) 105:1338–45. 10.1038/ajg.2009.719 20068559

[23] Bae JC Lee WY Yoon KH Park JY Son HS Han KA. Improvement of nonalcoholic fatty liver disease with carnitine-orotate complex in type 2 diabetes (CORONA): a randomized controlled trial. Diabetes Care. (2015) 38:1245–52. 10.2337/dc14-2852 25877813

[24] Ahmad MI Ijaz MU Hussain M Haq IU Zhao D Li C. High-fat proteins drive dynamic changes in gut microbiota, hepatic metabolome, and endotoxemia-TLR-4-NFkappaB-mediated inflammation in mice. J Agric Food Chem. (2020) 68:11710–25. 10.1021/acs.jafc.0c02570 33034193

[25] Vogel A Van Den Berg IE Al-Dhalimy M Groopman J Ou CN Ryabinina O. Chronic liver disease in murine hereditary tyrosinemia type 1 induces resistance to cell death. Hepatology. (2004) 39:433–43. 10.1002/hep.20077 14767996

[26] Li L Shi M Salerno S Tang M Guo F Liu J. Microbial and metabolomic remodeling by a formula of Sichuan dark tea improves hyperlipidemia in apoE-deficient mice. PLoS One. (2019) 14:e0219010. 10.1371/journal.pone.0219010 31269076 PMC6608967

[27] Kalyesubula M Mopuri R Asiku J Rosov A Yosefi S Edery N. High-dose vitamin B1 therapy prevents the development of experimental fatty liver driven by overnutrition. Dis Model Mech. (2021) 14. 10.1242/dmm.048355 33608323 PMC7988776

[28] Vahid F Hekmatdoost A Mirmajidi S Doaei S Rahmani D Faghfoori Z. Association between index of nutritional quality and nonalcoholic fatty liver disease: the role of vitamin D and B group. Am J Med Sci. (2019) 358:212–8. 10.1016/j.amjms.2019.06.008 31326093

[29] Naudin CR Maner-Smith K Owens JA Wynn GM Robinson BS Matthews JD. Lactococcus lactis subspecies cremoris elicits protection against metabolic changes induced by a western-style diet. Gastroenterology. (2020) 159:639–651. e635. 10.1053/j.gastro.2020.03.010 32169430

[30] Gradisteanu Pircalabioru G Ilie I Oprea L Picu A Petcu LM Burlibasa L. Microbiome, mycobiome and related metabolites alterations in patients with metabolic syndrome—A pilot study. Metabolites. (2022) 12. 10.3390/metabo12030218 35323661 PMC8951583

[31] Leylabadlo HE Ghotaslou R Feizabadi MM Farajnia S Moaddab SY Ganbarov K. The critical role of Faecalibacterium prausnitzii in human health: an overview. Microb Pathog. (2020) 149:104344. 10.1016/j.micpath.2020.104344 32534182

[32] Lu P Yamaguchi Y Fulton WB Wang S Zhou Q Jia HJr. Maternal aryl hydrocarbon receptor activation protects newborns against necrotizing enterocolitis. Nat Commun. (2021) 12:1042. 10.1038/s41467-021-21356-4 33589625 PMC7884836

